# Supplementary material for: A Likelihood Approach for Real-Time Calibration of Stochastic Compartmental Epidemic Models
Source: PLoS Comput Biol. 2017 Jan 17;13(1):e1005257. doi: 10.1371/journal.pcbi.1005257 (PMC5240920; doi:10.1371/journal.pcbi.1005257)
Supplement: S1 File — (TAR.GZ) [file pcbi.1005257.s014.tar.gz › HSPH_Online-SI-Revision/MSS10/n1-mild/n1-mild_NEW2_table4.pdf]

1	newly 3 weeks spec	6.	13.97	{1., 27.}	161.5	0.501944	newly 3 weeks cumul	40.	49.47	{14., 82.}	53.475	0.225818	newly 5 weeks spec	2.	9.2	{0., 18.}	400.	0.777547	newly 5 weeks cumul	43.	69.69	{21., 112.}	83.6977	0.281208	newly 51 weeks cumul	116.	97.89	{21., 189.}	45.0086	0.291039
2	newly 3 weeks spec	3.	2.61	{0., 6.}	101.667	0.864473	newly 3 weeks cumul	16.	12.7	{3., 27.}	54.25	0.341447	newly 5 weeks spec	4.	0.68	{0., 2.}	96.	1.41936	newly 5 weeks cumul	23.	14.63	{3., 30.}	60.1304	0.434262	newly 47 weeks cumul	28.	15.85	{3., 35.}	66.4643	0.503858
3	newly 3 weeks spec	13.	4.96	{0., 16.}	82.	1.31801	newly 3 weeks cumul	35.	32.32	{4., 67.}	62.3429	0.381067	newly 5 weeks spec	6.	1.98	{0., 7.}	99.6667	1.47142	newly 5 weeks cumul	61.	37.47	{4., 80.}	59.5902	0.49319	newly 56 weeks cumul	77.	43.24	{4., 107.}	66.5714	0.571914
4	newly 3 weeks spec	9.	9.92	{0., 29.}	119.778	1.09589	newly 3 weeks cumul	30.	35.02	{3., 82.}	84.5333	0.437107	newly 5 weeks spec	8.	4.62	{0., 17.}	110.75	1.44474	newly 5 weeks cumul	45.	47.62	{3., 121.}	88.1333	0.513809	newly 50 weeks cumul	300.	53.37	{3., 132.}	82.21	1.09232
5	newly 3 weeks spec	10.	2.71	{0., 7.}	86.1	1.3769	newly 3 weeks cumul	42.	23.29	{4., 48.}	52.3095	0.417987	newly 5 weeks spec	5.	0.51	{0., 1.}	97.	1.55117	newly 5 weeks cumul	52.	25.18	{4., 48.}	58.1538	0.488446	newly 52 weeks cumul	70.	25.25	{4., 48.}	65.1857	0.598453
6	newly 3 weeks spec	20.	22.94	{0., 49.}	75.9	0.520222	newly 3 weeks cumul	66.	89.77	{16., 178.}	72.9848	0.302201	newly 5 weeks spec	12.	10.8	{0., 27.}	76.5	0.752652	newly 5 weeks cumul	97.	117.07	{16., 221.}	62.3608	0.303171	newly 56 weeks cumul	201.	132.45	{16., 238.}	47.1095	0.380131
7	newly 3 weeks spec	20.	8.19	{0., 24.}	71.25	1.11103	newly 3 weeks cumul	60.	84.25	{11., 159.}	83.45	0.347071	newly 5 weeks spec	5.	0.7	{0., 1.}	100.4	1.54595	newly 5 weeks cumul	86.	87.82	{11., 174.}	62.0233	0.357267	newly 55 weeks cumul	126.	88.08	{11., 174.}	48.1587	0.386011
8	newly 3 weeks spec	10.	12.42	{2., 26.}	77.4	0.408374	newly 3 weeks cumul	44.	43.69	{12., 78.}	44.4773	0.236727	newly 5 weeks spec	12.	8.67	{0., 19.}	61.0833	0.62774	newly 5 weeks cumul	74.	63.05	{14., 119.}	43.527	0.271462	newly 47 weeks cumul	324.	101.63	{14., 193.}	68.6327	0.652893
9	newly 3 weeks spec	13.	7.28	{0., 17.}	62.7692	0.717727	newly 3 weeks cumul	57.	38.88	{9., 72.}	45.7544	0.337925	newly 5 weeks spec	5.	3.05	{0., 10.}	91.	1.03533	newly 5 weeks cumul	72.	47.14	{9., 92.}	48.6389	0.375141	newly 46 weeks cumul	74.	51.7	{9., 99.}	51.973	0.387304
10	newly 3 weeks spec	5.	11.65	{5., 18.}	140.6	0.35545	newly 3 weeks cumul	23.	45.97	{25., 65.}	103.435	0.293468	newly 5 weeks spec	1.	7.52	{1., 14.}	664.	0.824016	newly 5 weeks cumul	26.	62.82	{34., 90.}	144.	0.366126	newly 47 weeks cumul	26.	84.57	{44., 132.}	227.423	0.485278
11	newly 3 weeks spec	6.	16.82	{5., 28.}	193.	0.482388	newly 3 weeks cumul	21.	56.07	{25., 83.}	172.143	0.415999	newly 5 weeks spec	11.	12.41	{1., 24.}	65.1818	0.42282	newly 5 weeks cumul	43.	83.81	{34., 130.}	106.814	0.319115	newly 46 weeks cumul	315.	146.44	{38., 253.}	53.5111	0.436363
12	newly 3 weeks spec	9.	9.5	{0., 28.}	116.444	1.07831	newly 3 weeks cumul	42.	44.88	{6., 110.}	73.1429	0.397961	newly 5 weeks spec	9.	3.58	{0., 11.}	102.444	1.58983	newly 5 weeks cumul	57.	54.77	{6., 132.}	75.386	0.460937	newly 47 weeks cumul	206.	62.84	{6., 157.}	71.0097	0.806838
13	newly 3 weeks spec	6.	4.95	{0., 11.}	70.5	0.615939	newly 3 weeks cumul	20.	20.46	{6., 36.}	51.1	0.26135	newly 5 weeks spec	0.1	2.37	{0., 7.}	2366.	0.781567	newly 5 weeks cumul	21.	25.99	{6., 47.}	69.381	0.303394	newly 48 weeks cumul	26.	31.57	{6., 67.}	75.8077	0.341193
14	newly 3 weeks spec	4.	8.73	{1., 17.}	156.25	0.507199	newly 3 weeks cumul	23.	36.65	{13., 62.}	79.1739	0.260746	newly 5 weeks spec	3.	5.24	{0., 13.}	148.667	0.716902	newly 5 weeks cumul	29.	48.58	{14., 85.}	90.2759	0.294922	newly 52 weeks cumul	29.	59.98	{14., 119.}	129.586	0.358108
15	newly 3 weeks spec	19.	11.05	{1., 25.}	60.8947	0.605054	newly 3 weeks cumul	57.	50.69	{14., 87.}	43.1053	0.254098	newly 5 weeks spec	13.	5.16	{0., 17.}	72.4615	1.11036	newly 5 weeks cumul	84.	64.21	{14., 109.}	44.0595	0.295827	newly 52 weeks cumul	175.	73.45	{14., 137.}	59.32	0.508603
16	newly 3 weeks spec	24.	12.26	{6., 19.}	49.25	0.3464	newly 3 weeks cumul	51.	35.78	{26., 47.}	30.7451	0.173615	newly 5 weeks spec	13.	12.48	{4., 22.}	44.	0.266716	newly 5 weeks cumul	79.	60.07	{39., 83.}	27.4051	0.158482	newly 50 weeks cumul	230.	200.09	{71., 302.}	33.4304	0.210961
17	newly 3 weeks spec	19.	12.84	{1., 26.}	55.0526	0.502823	newly 3 weeks cumul	42.	44.05	{11., 78.}	52.5	0.266803	newly 5 weeks spec	16.	9.	{0., 22.}	62.75	0.750188	newly 5 weeks cumul	69.	64.37	{13., 120.}	50.6232	0.296998	newly 53 weeks cumul	203.	102.95	{13., 213.}	54.3399	0.487273
18	newly 3 weeks spec	17.	11.05	{1., 23.}	56.2941	0.520285	newly 3 weeks cumul	55.	35.91	{14., 60.}	42.0182	0.286695	newly 5 weeks spec	17.	7.36	{0., 15.}	62.2353	0.791272	newly 5 weeks cumul	88.	52.03	{17., 86.}	46.1023	0.339168	newly 46 weeks cumul	176.	83.69	{18., 170.}	55.1875	0.470809
19	newly 3 weeks spec	22.	6.48	{0., 17.}	72.0909	1.04589	newly 3 weeks cumul	64.	30.51	{6., 55.}	55.7656	0.464239	newly 5 weeks spec	12.	2.62	{0., 8.}	82.3333	1.41023	newly 5 weeks cumul	88.	37.69	{6., 78.}	59.0341	0.531971	newly 53 weeks cumul	114.	42.22	{6., 94.}	63.6491	0.612083
20	newly 3 weeks spec	10.	10.78	{0., 24.}	80.4	0.673517	newly 3 weeks cumul	38.	44.86	{9., 85.}	64.5789	0.299465	newly 5 weeks spec	11.	4.95	{0., 13.}	76.4545	1.14615	newly 5 weeks cumul	53.	57.08	{9., 119.}	63.7358	0.337131	newly 50 weeks cumul	69.	65.76	{9., 142.}	62.7246	0.377828
21	newly 3 weeks spec	11.	5.91	{0., 17.}	76.8182	1.02954	newly 3 weeks cumul	47.	35.44	{6., 68.}	49.4043	0.354428	newly 5 weeks spec	5.	2.24	{0., 10.}	101.6	1.36331	newly 5 weeks cumul	63.	40.99	{6., 87.}	52.4921	0.419807	newly 50 weeks cumul	68.	44.07	{6., 96.}	55.4265	0.444438
22	newly 3 weeks spec	16.	22.09	{9., 36.}	57.5625	0.202121	newly 3 weeks cumul	38.	64.52	{36., 99.}	74.	0.22151	newly 5 weeks spec	16.	23.06	{8., 35.}	64.125	0.230447	newly 5 weeks cumul	74.	110.2	{57., 162.}	58.2703	0.190538	newly 52 weeks cumul	332.	332.26	{162., 498.}	31.2289	0.157656
23	newly 3 weeks spec	14.	9.51	{0., 20.}	61.6429	0.696272	newly 3 weeks cumul	59.	41.05	{8., 75.}	44.1864	0.33349	newly 5 weeks spec	8.	3.69	{0., 11.}	76.375	1.03824	newly 5 weeks cumul	90.	50.8	{8., 94.}	48.6	0.412087	newly 47 weeks cumul	150.	56.92	{8., 113.}	62.84	0.587144
24	newly 3 weeks spec	16.	13.75	{3., 26.}	47.5625	0.346473	newly 3 weeks cumul	68.	48.51	{20., 77.}	35.9265	0.226937	newly 5 weeks spec	13.	9.75	{0., 21.}	52.2308	0.551851	newly 5 weeks cumul	98.	69.74	{29., 113.}	36.3878	0.237762	newly 49 weeks cumul	197.	113.55	{38., 196.}	47.3046	0.353651
25	newly 3 weeks spec	13.	16.6	{8., 26.}	53.0769	0.276819	newly 3 weeks cumul	29.	55.63	{29., 77.}	98.3103	0.290516	newly 5 weeks spec	8.	14.75	{4., 25.}	109.625	0.409847	newly 5 weeks cumul	44.	86.01	{39., 127.}	104.977	0.311896	newly 46 weeks cumul	191.	182.42	{56., 286.}	33.2984	0.202237
26	newly 3 weeks spec	5.	10.51	{2., 23.}	139.	0.416299	newly 3 weeks cumul	24.	37.24	{17., 57.}	69.5	0.231587	newly 5 weeks spec	1.	7.14	{0., 16.}	646.	0.838624	newly 5 weeks cumul	34.	52.64	{21., 87.}	74.6471	0.250313	newly 49 weeks cumul	44.	73.98	{21., 132.}	93.2727	0.300713
27	newly 3 weeks spec	19.	11.5	{0., 27.}	58.9474	0.663968	newly 3 weeks cumul	39.	42.86	{12., 77.}	54.1538	0.252593	newly 5 weeks spec	10.	7.35	{0., 20.}	82.5	0.967208	newly 5 weeks cumul	66.	60.09	{13., 118.}	51.6818	0.303314	newly 46 weeks cumul	107.	78.73	{13., 163.}	53.1682	0.382849
28	newly 3 weeks spec	12.	24.24	{7., 40.}	118.5	0.386271	newly 3 weeks cumul	35.	70.3	{34., 100.}	107.486	0.296543	newly 5 weeks spec	8.	24.19	{5., 41.}	220.125	0.572295	newly 5 weeks cumul	50.	119.63	{54., 170.}	145.82	0.373157	newly 52 weeks cumul	232.	334.89	{95., 505.}	69.5129	0.279167
29	newly 3 weeks spec	13.	8.15	{0., 18.}	63.6154	0.78291	newly 3 weeks cumul	47.	39.82	{7., 77.}	46.2979	0.312325	newly 5 weeks spec	5.	2.82	{0., 11.}	96.8	1.18372	newly 5 weeks cumul	61.	47.3	{7., 94.}	48.1311	0.356016	newly 49 weeks cumul	61.	50.86	{7., 104.}	52.8197	0.368711
30	newly 3 weeks spec	22.	10.61	{0., 23.}	60.3182	0.711651	newly 3 weeks cumul	51.	42.72	{9., 79.}	44.7059	0.280515	newly 5 weeks spec	11.	6.13	{0., 17.}	68.4545	0.855591	newly 5 weeks cumul	78.	57.03	{9., 104.}	45.859	0.332574	newly 53 weeks cumul	111.	71.4	{9., 156.}	50.6126	0.400992
31	newly 3 weeks spec	4.	7.33	{1., 16.}	124.75	0.451081	newly 3 weeks cumul	23.	34.75	{14., 55.}	67.6087	0.233696	newly 5 weeks spec	0.1	3.92	{0., 9.}	3882.	1.13168	newly 5 weeks cumul	29.	43.86	{17., 68.}	70.8966	0.250148	newly 51 weeks cumul	29.	51.18	{17., 86.}	95.6552	0.30088
32	newly 3 weeks spec	21.	10.42	{0., 19.}	61.2381	0.629346	newly 3 weeks cumul	44.	34.22	{12., 54.}	44.6818	0.260269	newly 5 weeks spec	15.	8.15	{0., 18.}	61.2667	0.781976	newly 5 weeks cumul	72.	51.77	{12., 84.}	44.5139	0.302506	newly 49 weeks cumul	281.	113.8	{12., 237.}	59.5943	0.580223
33	newly 3 weeks spec	13.	23.95	{11., 33.}	92.8462	0.278252	newly 3 weeks cumul	55.	85.76	{58., 109.}	59.3455	0.197083	newly 5 weeks spec	7.	15.35	{6., 25.}	130.714	0.353164	newly 5 weeks cumul	72.	120.7	{74., 161.}	71.3333	0.225902	newly 50 weeks cumul	85.	173.08	{92., 234.}	107.882	0.305636
34	newly 3 weeks spec	12.	7.61	{0., 18.}	63.5833	0.667659	newly 3 weeks cumul	36.	28.05	{5., 50.}	51.6389	0.328497	newly 5 weeks spec	15.	4.77	{0., 13.}	72.8667	1.14274	newly 5 weeks cumul	60.	38.99	{6., 78.}	53.1167	0.404717	newly 49 weeks cumul	138.	55.99	{6., 127.}	63.3116	0.62222
35	newly 3 weeks spec	15.	27.65	{18., 37.}	85.4	0.25333	newly 3 weeks cumul	76.	93.04	{72., 114.}	26.7368	0.102214	newly 5 weeks spec	15.	18.84	{9., 28.}	44.2667	0.167282	newly 5 weeks cumul	115.	135.45	{101., 168.}	24.1652	0.0946127	newly 51 weeks cumul	153.	213.64	{144., 283.}	44.1046	0.154393
36	newly 3 weeks spec	2.	4.87	{0., 12.}	215.5	0.737691	newly 3 weeks cumul	28.	30.42	{6., 67.}	62.9286	0.306954	newly 5 weeks spec	0.1	1.34	{0., 5.}	1388.	0.41016	newly 5 weeks cumul	28.	34.78	{6., 77.}	75.2857	0.328389	newly 53 weeks cumul	28.	35.93	{6., 78.}	79.1786	0.335386
37	newly 3 weeks spec	11.	11.05	{0., 25.}	81.5455	0.700359	newly 3 weeks cumul	35.	44.07	{7., 81.}	67.5143	0.313774	newly 5 weeks spec	8.	5.44	{0., 15.}	83.	0.999796	newly 5 weeks cumul	53.	58.07	{7., 112.}	61.4151	0.34568	newly 49 weeks cumul	95.	74.59	{7., 157.}	57.5263	0.438336
38	newly 3 weeks spec	9.	4.34	{0., 15.}	85.1111	1.14681	newly 3 weeks cumul	41.	28.13	{5., 55.}	54.122	0.401069	newly 5 weeks spec	5.	0.71	{0., 3.}	91.	1.43936	newly 5 weeks cumul	58.	30.52	{5., 64.}	58.931	0.495079	newly 53 weeks cumul	65.	31.08	{5., 66.}	61.0769	0.532917
39	newly 3 weeks spec	13.	6.65	{0., 15.}	59.9231	0.715062	newly 3 weeks cumul	39.	28.12	{6., 48.}	42.4103	0.308835	newly 5 weeks spec	4.	3.57	{0., 10.}	103.75	0.968889	newly 5 weeks cumul	49.	36.93	{6., 62.}	45.7347	0.328798	newly 51 weeks cumul	78.	43.76	{6., 91.}	56.4359	0.461436
40	newly 3 weeks spec	11.	5.49	{0., 15.}	65.9091	0.856196	newly 3 weeks cumul	36.	39.67	{9., 69.}	51.9722	0.253458	newly 5 weeks spec	1.	2.3	{0., 9.}	258.	0.867944	newly 5 weeks cumul	43.	45.27	{9., 82.}	53.5116	0.277573	newly 54 weeks cumul	43.	47.83	{9., 92.}	58.7209	0.288272
41	newly 3 weeks spec	9.	9.58	{0., 25.}	105.111	0.924746	newly 3 weeks cumul	33.	60.22	{4., 138.}	129.333	0.460052	newly 5 weeks spec	5.	2.28	{0., 7.}	98.8	1.28109	newly 5 weeks cumul	42.	67.02	{4., 145.}	111.81	0.463284	newly 55 weeks cumul	48.	70.63	{4., 162.}	103.396	0.468573
42	newly 3 weeks spec	0.1	2.07	{0., 8.}	2126.	0.392619	newly 3 weeks cumul	0.1	15.18	{2., 36.}	15 080.	1.96188	newly 5 weeks spec	0.1	0.51	{0., 0.}	598.	0.102455	newly 5 weeks cumul	0.1	16.47	{2., 42.}	16 370.	1.97237	newly 54 weeks cumul	0.1	17.21	{2., 42.}	17 110.	1.97541
43	newly 3 weeks spec	2.	6.28	{0., 17.}	266.	0.666582	newly 3 weeks cumul	19.	31.34	{8., 58.}	91.6842	0.296702	newly 5 weeks spec	2.	1.41	{0., 4.}	112.5	0.967013	newly 5 weeks cumul	24.	35.61	{8., 66.}	80.7083	0.287796	newly 56 weeks cumul	27.	36.91	{8., 70.}	73.5185	0.283401
44	newly 3 weeks spec	2.	4.55	{0., 14.}	235.5	0.93034	newly 3 weeks cumul	12.	27.67	{4., 67.}	161.583	0.400149	newly 5 weeks spec	0.1	1.53	{0., 7.}	1588.	0.358217	newly 5 weeks cumul	14.	32.1	{4., 79.}	166.286	0.416419	newly 48 weeks cumul	17.	33.41	{4., 85.}	141.824	0.410104
45	newly 3 weeks spec	13.	8.49	{0., 19.}	58.8462	0.591624	newly 3 weeks cumul	46.	36.34	{7., 71.}	44.5652	0.284703	newly 5 weeks spec	7.	4.07	{0., 13.}	81.8571	0.970767	newly 5 weeks cumul	63.	45.77	{9., 94.}	48.4286	0.326341	newly 46 weeks cumul	86.	53.26	{9., 118.}	53.5581	0.399548
46	newly 3 weeks spec	11.	8.82	{0., 25.}	85.8182	0.944002	newly 3 weeks cumul	54.	34.92	{5., 76.}	57.5185	0.443203	newly 5 weeks spec	9.	6.26	{0., 20.}	102.889	1.26115	newly 5 weeks cumul	77.	48.36	{5., 119.}	63.0909	0.520209	newly 53 weeks cumul	299.	64.16	{5., 171.}	79.291	0.981843
47	newly 3 weeks spec	14.	14.77	{4., 29.}	57.2143	0.312187	newly 3 weeks cumul	32.	43.36	{17., 72.}	57.8125	0.214222	newly 5 weeks spec	10.	11.35	{0., 23.}	74.9	0.481166	newly 5 weeks cumul	50.	67.17	{22., 119.}	61.18	0.233541	newly 51 weeks cumul	260.	138.15	{31., 279.}	51.7346	0.437647
48	newly 3 weeks spec	9.	27.78	{5., 38.}	226.222	0.616119	newly 3 weeks cumul	23.	93.19	{48., 115.}	313.087	0.61843	newly 5 weeks spec	3.	22.55	{0., 32.}	677.667	0.997616	newly 5 weeks cumul	34.	142.46	{70., 176.}	328.882	0.642853	newly 46 weeks cumul	83.	292.24	{70., 369.}	266.578	0.597551
49	newly 3 weeks spec	8.	21.64	{12., 32.}	171.	0.402108	newly 3 weeks cumul	37.	62.56	{44., 81.}	70.7027	0.221109	newly 5 weeks spec	3.	21.93	{10., 34.}	632.333	0.815437	newly 5 weeks cumul	43.	106.7	{71., 145.}	148.884	0.380222	newly 50 weeks cumul	190.	300.26	{206., 399.}	66.3368	0.226557
50	newly 3 weeks spec	19.	8.11	{1., 17.}	60.0526	0.59469	newly 3 weeks cumul	60.	31.11	{12., 51.}	49.4833	0.356678	newly 5 weeks spec	13.	4.94	{0., 11.}	67.8462	0.878997	newly 5 weeks cumul	91.	42.25	{13., 77.}	54.3407	0.419108	newly 47 weeks cumul	148.	56.76	{14., 101.}	63.	0.540678
